# Apolipoprotein D Expression Dynamics During Cuprizone-Induced Demyelination and Remyelination in a Mouse Model of Multiple Sclerosis

**DOI:** 10.3390/ijms26178692

**Published:** 2025-09-06

**Authors:** Eva Martínez-Pinilla, Nuria Rubio-Sardón, Gemma Fernández-García, Sandra Villar-Conde, Carlota Menéndez-Pérez, Jorge Tolivia, Eva del Valle, Ana Navarro

**Affiliations:** 1 Department of Morphology and Cell Biology, University of Oviedo, 33006 Oviedo, Spain; nuria199510@hotmail.com (N.R.-S.); villarsandra@uniovi.es (S.V.-C.); menendezperezcarlota@gmail.com (C.M.-P.); valleeva@uniovi.es (E.d.V.); anavarro@uniovi.es (A.N.); 2 Instituto de Neurociencias del Principado de Asturias (INEUROPA), 33006 Oviedo, Spain; 3 Instituto de Investigación Sanitaria del Principado de Asturias (ISPA), 33006 Oviedo, Spain; fernandezgemma@uniovi.es; 4 Department of Functional Biology, University of Oviedo, 33006 Oviedo, Spain; 5 Instituto Universitario de Oncología del Principado de Asturias (IUOPA), 33006 Oviedo, Spain

**Keywords:** apolipoprotein D, cuprizone, multiple sclerosis, neuroprotection, remyelination, oligodendrocytes, clozapine

## Abstract

Multiple sclerosis (MS) is a chronic, immune-mediated disease of the central nervous system (CNS) characterized by oligodendrocyte (OLG) degeneration, myelin loss, and impaired remyelination. Apolipoprotein D (Apo D), a glia-derived lipocalin, has emerged in recent decades as a neuroprotective molecule involved in lipid transport, oxidative stress regulation, and inflammation control during aging and neurodegenerative diseases like MS. However, its role in demyelination/remyelination dynamics remains poorly defined. In this study, we used the cuprizone (CPZ)-induced demyelination model in C57BL/6 mice to analyze Apo D expression patterns in the corpus callosum during de- and remyelination. We also assessed whether the atypical antipsychotic clozapine (CLO), previously shown to upregulate Apo D in vivo, could modulate its expression and influence myelin recovery in this pathological context. Using a combination of magnetic resonance imaging, Luxol fast blue staining, and double immunohistochemistry, we demonstrated that CPZ treatment for 3 or 6 weeks led to significant demyelination, hydrocephalus, and reduced motor cortex thickness, which were partially reversed after treatment cessation. Apo D expression in OLGs was significantly reduced by CPZ exposure, both at the protein level and in terms of immunoreactive cell counts, but was restored following treatment withdrawal. Notably, co-administration of CLO prevented the CPZ-induced reduction in Apo D expression in OLGs, although it did not attenuate myelin loss. In this way, our results reveal a strong correlation between Apo D expression and OLG/myelin integrity in vivo. While CLO did not exert remyelinating effects, it preserved Apo D levels under demyelinating conditions, suggesting a potential indirect neuroprotective mechanism. These findings support the relevance of Apo D in CNS myelin homeostasis and highlight its potential as a molecular target for therapeutic intervention in demyelinating diseases such as MS.

## 1. Introduction

Multiple sclerosis (MS) is an autoimmune, inflammatory, and neurodegenerative disease of the central nervous system (CNS), characterized by two main pathological processes: progressive oligodendrocyte (OLG) degeneration and myelin disruption around axons in both white and gray matter [1,2,3,4,5]. These processes manifest clinically as neurological symptoms, such as sensory disturbances, motor weakness, visual impairment, and coordination deficits [6,7,8]. Under non-pathological conditions, demyelination is followed by spontaneous remyelination, a process in which new myelin is generated in an attempt to recoat axons and restore their function [9,10,11]. In the CNS, remyelination is primarily driven by the proliferation and differentiation of resident oligodendroglial progenitor cells (OPCs) and neural stem cells (NSCs) within the lesioned areas [9,12,13,14]. However, in MS, this OLG-dependent regenerative process is often inefficient, ultimately leading to irreversible neurodegeneration for which no curative therapy currently exists [9,13,14,15,16]. Thus, understanding the molecular and cellular mechanisms underlying demyelination and remyelination is crucial for developing effective therapeutic strategies. To achieve this, the use of experimental models that mimic key aspects of MS and other human myelin-related diseases is essential.

In recent decades, exposing adult mice to a cuprizone (CPZ)-enriched diet has become a well-established and reversible model for studying the demyelination and remyelination processes involved in MS [17,18,19]. Several studies have demonstrated that this copper chelator induces acute OLG degeneration and extensive demyelination over time, particularly in the corpus callosum, hippocampus, and other regions of the rodent CNS, followed by partial remyelination once CPZ treatment ceases [20,21,22,23]. Although the complex biology of remyelination has yet to be fully elucidated, it is known that multiple intracellular and extracellular molecules play crucial roles in the repair of lesions [13,24,25,26,27,28]. Among these, apolipoprotein D (Apo D) is a key component of this intricate process.

Apo D is a member of the lipocalin family, known for binding and transporting various small hydrophobic ligands, such as cholesterol and arachidonic acid, under both physiological and pathological conditions [29,30,31]. This function is attributed to its unique tertiary structure, which forms a lipid-specific ligand-binding pocket [32,33,34,35,36]. Notably, this 29 kDa glycoprotein exhibits a distinctive amino acid composition, including three methionine residues (Met49, Met93, and Met157) and an additional cysteine residue (Cys116) with a thiol group [37,38] that have been linked to the ability of Apo D to bind and reduce oxidized lipids, and to inhibit the radical propagation of lipid hydroperoxides [39,40,41]. In this sense, a range of biological effects has been described for this apolipoprotein. For example, it plays a crucial role in maintaining cellular homeostasis by stabilizing biological membranes, ensuring proper organelle function, and regulating vesicle trafficking [42,43]. Additionally, it serves as an antioxidant, participating in protective mechanisms against oxidative stress [43,44,45,46,47]. More importantly, Apo D has been shown to modulate inflammatory responses by attenuating the production of signaling mediators, thereby limiting both the extent and duration of inflammation [48,49,50]. These functions are particularly relevant in the nervous system (NS), where it is now recognized as an endogenous molecule with neuroprotective properties that become particularly relevant in response to various pathological conditions [45,51]. It has been demonstrated that periventricular cells, pericytes, astrocytes, and myelinating glia (i.e., OLGs and Schwann cells) produce and accumulate high levels of Apo D in the cytosol. Once secreted, Apo D can be reabsorbed by neurons in a paracrine manner [16,52,53], contributing to neuronal homeostasis and the compaction of the myelin extracellular leaflet during development and differentiation [49,54,55]. Interestingly, Apo D expression is upregulated in neural cells and cerebrospinal fluid (CSF) during aging [56,57,58], and in response to various CNS injuries [48]. In several neurodegenerative diseases, including Alzheimer’s disease (AD) [55,59], MS [16,60], and Niemann–Pick disease [61] elevated Apo D levels have also observed. In addition, the increased expression of this apolipoprotein in psychiatric disorders has led to its proposal as a potential biomarker for diagnosis [39,62,63,64]. In this context, Apo D has been linked to the effects of clozapine (CLO), an atypical antipsychotic used in bipolar disorder and schizophrenia which has gained attention beyond its psychiatric indications due to its reported neuroprotective properties. Studies indicate that continuous CLO treatment for two weeks increases Apo D expression at both transcriptional and protein levels, suggesting a potential role for this apolipoprotein in CLO-induced neuroprotection and neuronal homeostasis [65,66].

Moreover, the neuroprotective role of Apo D has also been investigated in the context of demyelination and remyelination in both the CNS and peripheral nervous system (PNS). Experimental studies have shown that its local synthesis increases following peripheral nerve injury, where it is essential for the proper clearance and recycling of damaged nerve fibers via lysosomal-dependent degradation, as well as for the reconstruction of myelin sheaths [42,67,68,69]. In this scenario, Apo D plays a key role in controlling inflammation and facilitating tissue recovery in the lesioned areas [49,54]. In MS, proteomic analyses have revealed increased levels of Apo D in the CSF of patients [60,70]. Remarkably, a pioneering study analyzing Apo D expression in human MS lesions found that while reactive astrocytes and mature OLGs are the primary producers of this protein, its levels are markedly low in all sclerosis plaques, particularly in inactive areas. However, its expression recovers in regions undergoing remyelination [16], suggesting a potential contribution in the regenerative response.

This study aims to investigate the dynamics of Apo D expression during demyelination and remyelination in the CPZ-induced MS model, and to assess the potential modulatory effect of CLO on this expression. Through this approach, we seek to clarify the role of Apo D in myelin integrity and its relevance as a molecular target in demyelinating diseases such as MS.

## 2. Results

### 2.1. Cuprizone Administration Induces Demyelination and Other Brain Alterations, Which Are Reversed upon Treatment Discontinuation

**Figure 1:**
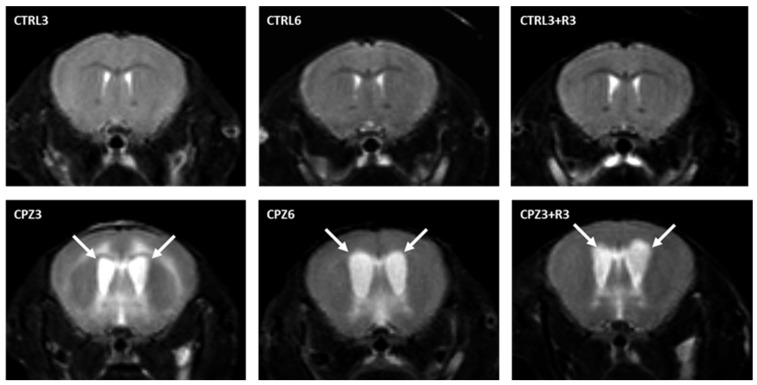
T2-weighted coronal magnetic resonance images at the level of the anterior commissure from mouse brains: control, treated with cuprizone for 3 weeks (CPZ3), 6 weeks (CPZ6), and 3 weeks followed by a 3-week recovery period (CPZ3+R3). Arrows indicate hyperintense areas.

**Figure 2:**
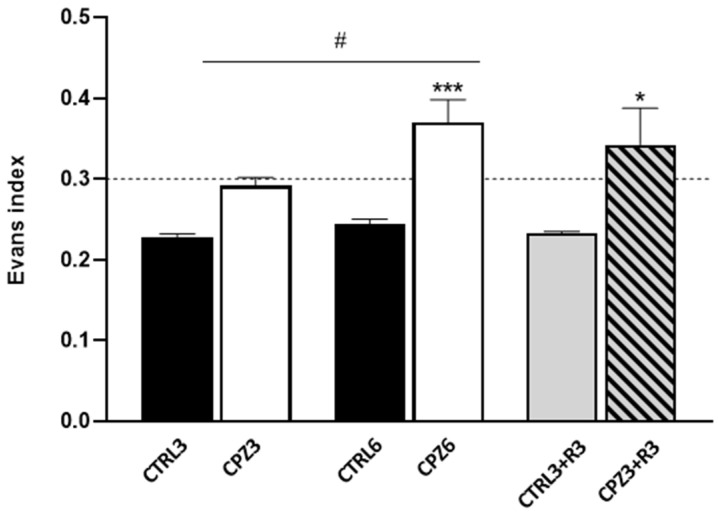
Evans index indicating the degree of hydrocephalus in mice treated with cuprizone for 3 weeks (CPZ3), 6 weeks (CPZ6), and 3 weeks followed by a 3-week recovery period (CPZ3+R3), along with their respective controls. Data are presented as mean ± SEM; *n* = 6 per group. Statistical analysis was performed using two-way ANOVA followed by Bonferroni’s multiple comparisons test. * *p* < 0.05; *** *p* < 0.001 versus control; # *p* < 0.05 versus CPZ3.

First and to investigate the effects of CPZ on the mouse brain, densitometric analyses of magnetic resonance imaging (MRI) were performed at the beginning and end of the treatment. T2-weighted MRI images were used to study the degree of demyelination but also allowed the detection of hydrocephalus in CPZ-treated mice (Figure 1). On one hand, hyperintense areas observed in the images, along with brain flattening, suggested a higher water content in the parenchyma. An increased intensity was noted at the level of the ventricles and in the surrounding nervous tissue in mice treated with CPZ for 3 weeks (CPZ3) compared to controls, regardless of recovery time (Figure 1; arrows). However, this finding was more pronounced in mice treated with CPZ for 6 weeks (CPZ6). On the other hand, the hydrocephalus was further demonstrated using the Evans index, calculated as the ratio of brain diameter to inter-ventricular distance. In fact, CPZ6 mice and those treated with CPZ for 3 weeks followed by a 3-week recovery period (CPZ3+R3) showed Evans ratios above 0.3, significantly higher than their respective controls (Figure 2).

**Figure 3:**
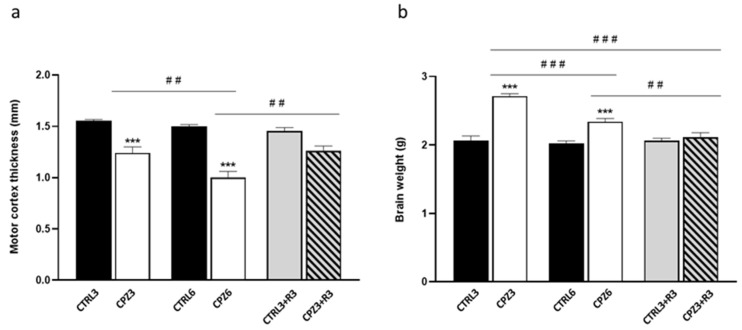
Motor cortex thickness (**a**) and brain weight (**b**) in mice treated with cuprizone for 3 weeks (CPZ3), 6 weeks (CPZ6), and 3 weeks followed by a 3-week recovery period (CPZ3+R3), along with their controls. Data are presented as mean ± SEM; *n* = 6 per group. Statistical analysis was performed using a two-way ANOVA followed by Bonferroni’s multiple comparisons test. *** *p* < 0.001 versus control; ## *p* < 0.01; ### *p* < 0.001 versus cuprizone. g: grams; mm: millimeters.

Next, based on the MRI findings, we investigated whether CPZ treatment also affected the motor cortex thickness and brain weight. As shown in Figure 3a, CPZ3 and CPZ6 mice exhibited a significant reduction in motor cortex thickness compared to untreated ones. Notably, this reduction was greater in CPZ6 mice (0.5 mm) than in CPZ3 mice (0.3 mm). Interestingly, CPZ3+R3 mice displayed motor cortex thickness similar to controls, suggesting that CPZ-induced changes are reversible upon treatment discontinuation. Regarding post-mortem brain weight, CPZ3 and CPZ6 mice had significantly higher brain weights than controls, but no differences were observed in mice after a 3-week recovery period (Figure 3b).

**Figure 4:**
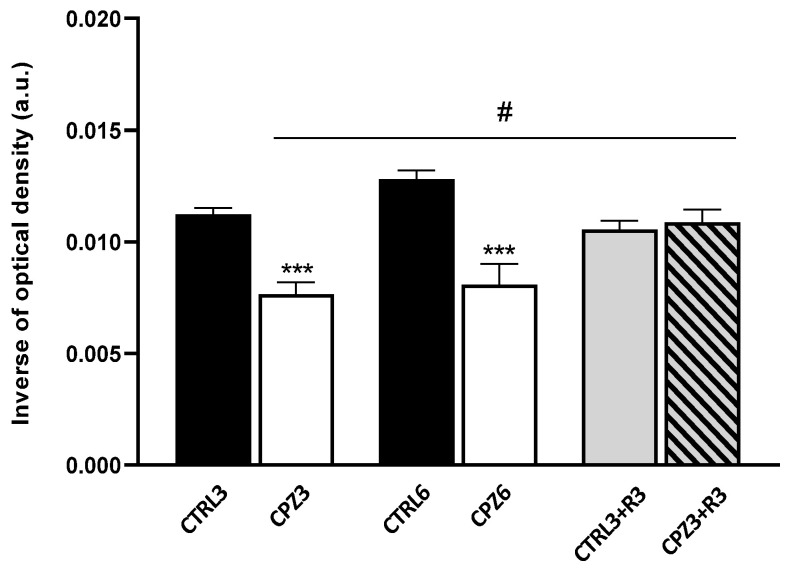
Quantification of demyelination degree in the corpus callosum of mice treated with cuprizone for 3 weeks (CPZ3), 6 weeks (CPZ6), and 3 weeks followed by 3 weeks of recovery (CPZ3+R3), along with their controls. Data are presented as mean ± SEM of inverse optical density from T2-weighted MRI images; *n* = 6 per group. Statistical analysis was performed using a two-way ANOVA followed by Bonferroni’s multiple comparisons test. *** *p* < 0.001 versus control; # *p* < 0.05 versus CPZ3. a.u.: arbitrary units.

The degree of demyelination induced by CPZ, a well-established effect in this murine model of cytotoxicity [71], was also assessed. Given that white matter myelin bundles are more intense than gray matter, the hyperintense signal detected in the corpus callosum (at the anterior commissure level) was quantified from T2-weighted MRI images (see Figure 1). The results revealed a significant decrease in myelin content in both CPZ3 and CPZ6 mice (Figure 4). However, MRI images showed no changes in optical density 3 weeks after CPZ treatment suspension compared to controls, suggesting that experimentally induced demyelination in this animal model is followed by a remyelination process.

**Figure 5:**
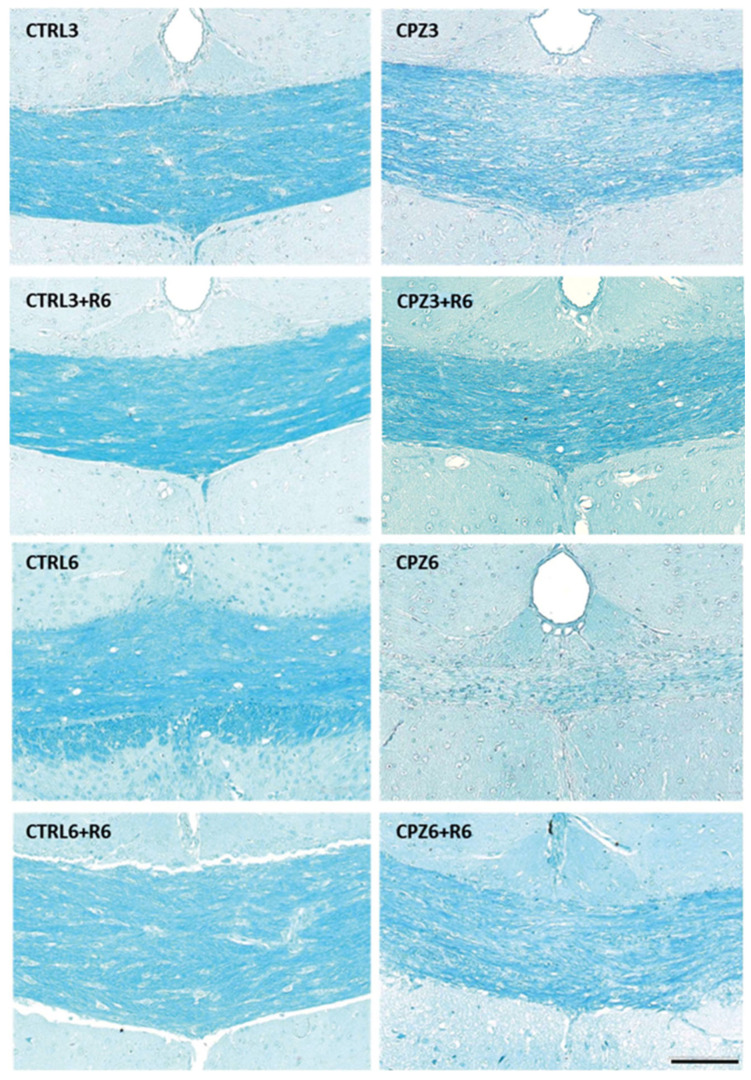
Representative images of coronal sections of the corpus callosum (at the 0–0.3 mm Bregma level), stained with Luxol Fast Blue, from brains of control mice and those treated with cuprizone for 3 weeks (CPZ3), 3 weeks followed by 6 weeks of recovery (CPZ3+R6), 6 weeks (CPZ6), and 6 weeks followed by 6 weeks of recovery (CPZ6+R6). Scale bar: 100 µm.

**Figure 6:**
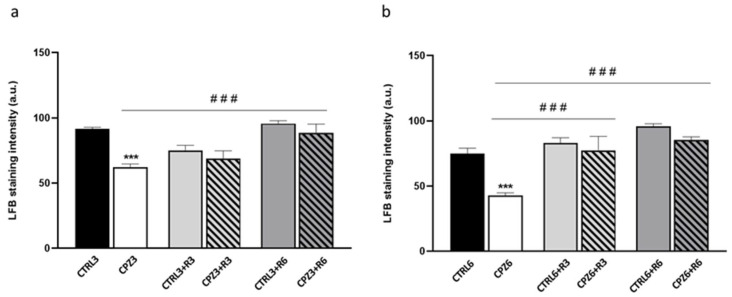
Densitometric quantification of myelin staining with Luxol Fast Blue (LFB) in the corpus callosum of mice treated with cuprizone for (**a**) 3 weeks (CPZ3), 3 weeks followed by 3 weeks of recovery (CPZ3+R3), and 3 weeks followed by 6 weeks of recovery (CPZ3+R6), along with their controls; and (**b**) 6 weeks (CPZ6), 6 weeks followed by 3 weeks of recovery (CPZ6+R3), and 6 weeks followed by 6 weeks of recovery (CPZ6+R6), along with their controls. Data are presented as mean staining intensity ± SEM; *n* = 6 per group. Statistical analysis was performed using a two-way ANOVA followed by Bonferroni’s multiple comparisons test. *** *p* < 0.001 versus control; ### *p* < 0.001 versus cuprizone. a.u.: arbitrary units.

To confirm the demyelination observed in MRI images, histological analysis was performed on mouse brain sections stained with Luxol fast blue (LFB). As shown in Figure 5, the blue staining intensity decreased in the corpus callosum of CPZ-treated mice, especially in CPZ6 mice. Similarly to MRI observations, discontinuation of CPZ treatment triggered a remyelination process that peaked at 6 weeks of recovery in both CPZ3 and CPZ6 mice; the intensity of blue labeling increased to levels like those of controls (Figure 5). Densitometric quantification of the LFB histochemical signal confirmed the previous observations, demonstrating a significant myelin loss after 3 and 6 weeks of CPZ treatment, which was reversed following 3- or 6-week recovery periods (Figure 6).

### 2.2. Cuprizone Administration Reduces Apolipoprotein D Expression in the Corpus Callosum, Which Recovers After Treatment Discontinuation

**Figure 7:**
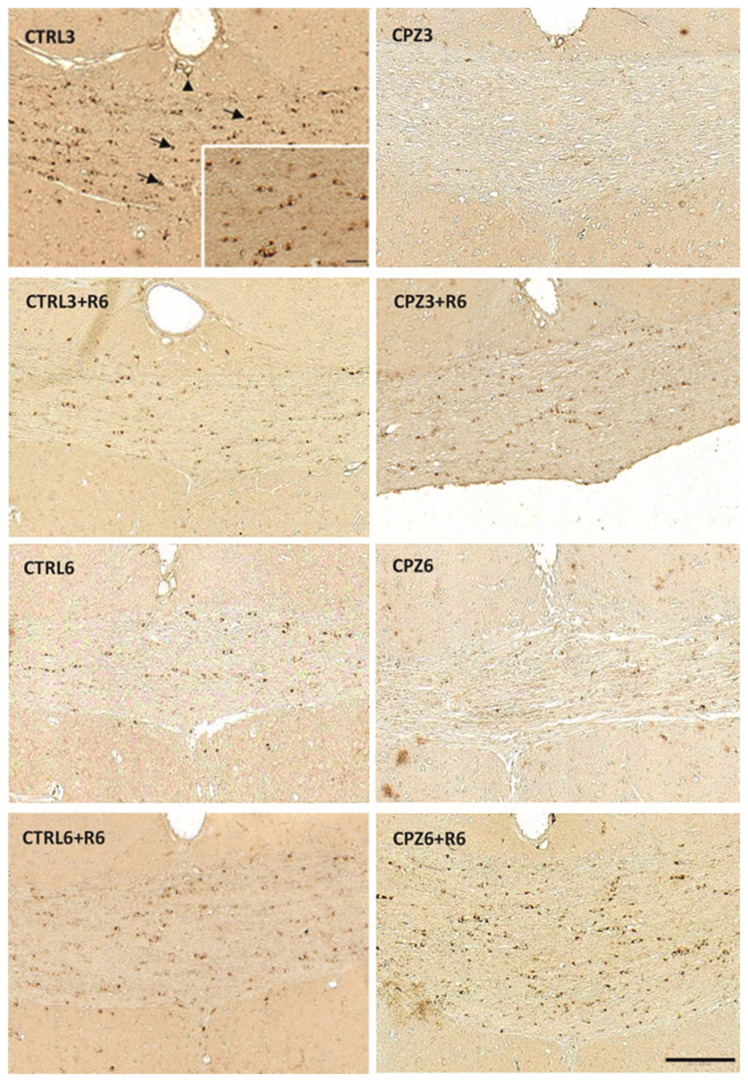
Representative images of Apolipoprotein D (Apo D) expression in coronal sections of the corpus callosum (0–0.3 mm Bregma level) from brains of control mice and those treated with cuprizone for 3 weeks (CPZ3), 3 weeks followed by 6 weeks of recovery (CPZ3+R6), 6 weeks (CPZ6), 6 weeks followed by 6 weeks of recovery (CPZ6+R6). Arrows: oligodendrocytes. Arrowhead: pericytes. Scale bar: 100 µm. Detail: 83×; scale bar 20 μm.

After characterizing the effects of CPZ on the brain in a murine model of MS induced by cytotoxicity, we evaluated Apo D expression in the corpus callosum by immunohistochemistry and double immunohistochemistry. Control mice showed clear Apo D staining in the cytoplasm of OLGs, with more intense labeling around the nucleus and extending into their projections (Figure 7; arrows). An evident signal was also detected in some pericytes near blood vessels (Figure 7; arrowhead). Interestingly, Apo D expression was nearly abolished in the corpus callosum of mice treated with CPZ for 3 and 6 weeks. However, discontinuation of CPZ treatment led to a recovery of oligodendrocytic Apo D expression to control levels (Figure 7).

**Figure 8:**
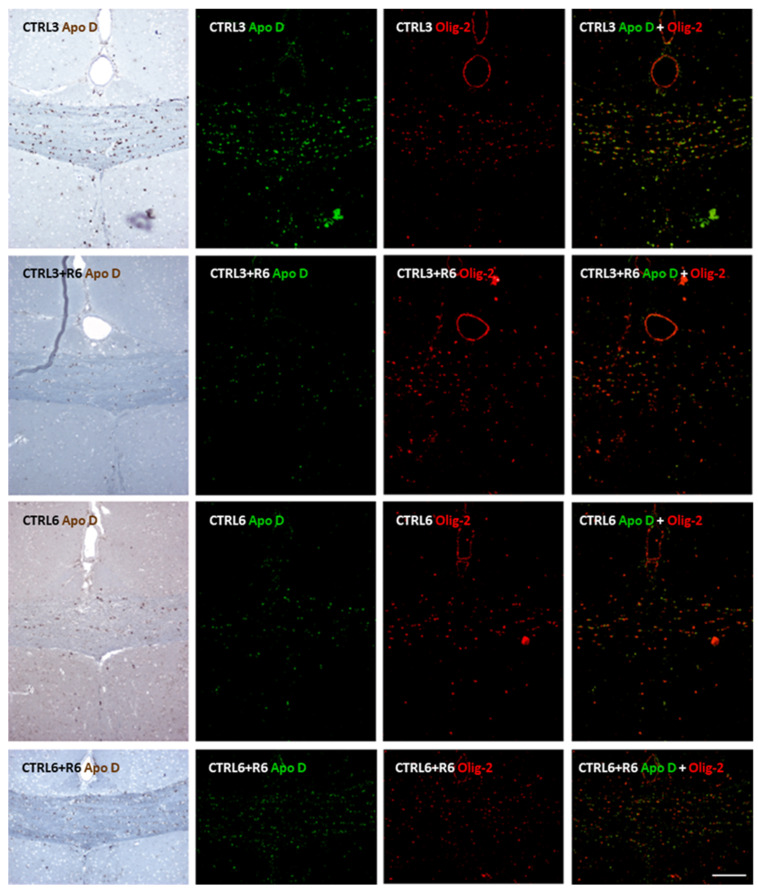
Representative confocal images showing double immunohistochemistry for Apolipoprotein D (Apo D; green) and Olig-2 (red) in the corpus callosum of the control mice for those treated with cuprizone for 3 weeks (CTRL3), 3 weeks followed by 6 weeks of recovery (CTRL3+R6), 6 weeks (CTRL6), and 6 weeks followed by 6 weeks of recovery (CTRL6+R6). Merged images reveal clear colocalization (yellow) in multiple cells, indicating that Apo D is expressed in Olig2-positive oligodendrocytes under basal conditions. Scale bar: 100 µm.

**Figure 9:**
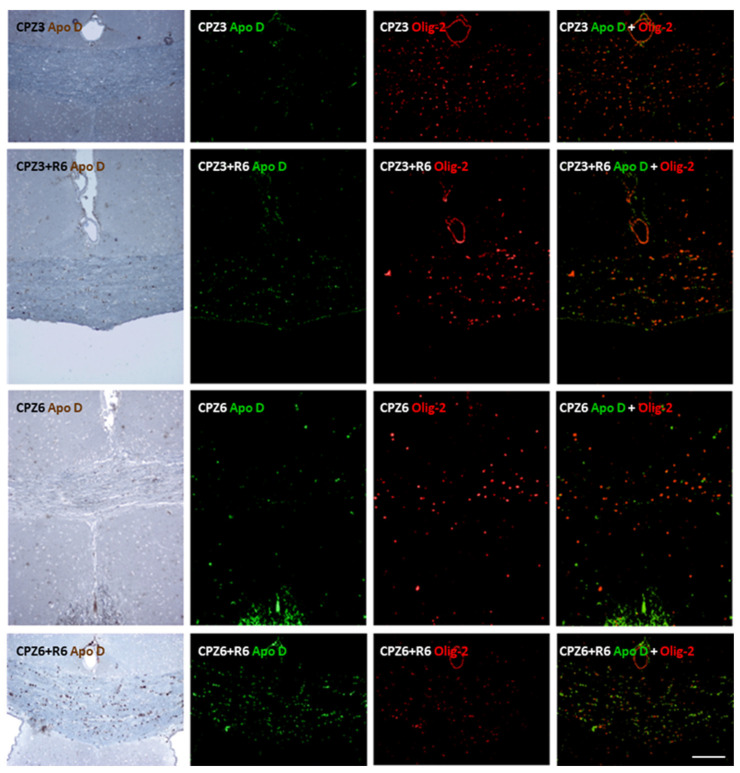
Representative confocal images showing double immunohistochemistry for Apolipoprotein D (Apo D; green) and Olig-2 (red) in the corpus callosum of mice treated with cuprizone for 3 weeks (CPZ3), 3 weeks followed by 6 weeks of recovery (CPZ3+R6), 6 weeks (CPZ6), and 6 weeks followed by 6 weeks of recovery (CPZ6+R6). Merged images reveal clear colocalization (yellow) in multiple cells, indicating that Apo D is expressed in Olig2-positive oligodendrocytes under demyelinating conditions. Scale bar: 100 µm.

To further validate that Apo D is specifically expressed in OLGs, we performed double immunohistochemistry using antibodies against Apo D and Olig-2, a well-established marker for oligodendroglial lineage cells. Representative images of the corpus callosum from all experimental groups are shown in Figure 8 and Figure 9. Colocalization of Apo D (green) and Olig-2 (red) was evident in several cells (yellow in merged images), confirming the oligodendroglial identity of Apo D-positive cells in this brain region. Furthermore, to rule out the possibility that Apo D expression in the corpus callosum could be attributed to astrocytes, we performed an additional double immunohistochemical staining using GFAP as a marker of astrocytic cells. As shown in Supplementary Figure S1, no significant colocalization of Apo D and GFAP signals were observed, indicating that Apo D-positive cells are distinct from the astrocytic population. These results reinforce our previous identification of Apo D-expressing cells as mature OLGs.

**Figure 10:**
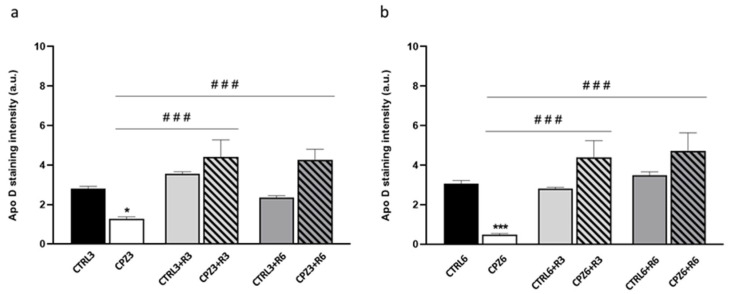
Densitometric quantification of Apolipoprotein D (Apo D) immunostaining in the corpus callosum of mice treated with cuprizone for (**a**) 3 weeks (CPZ3), 3 weeks followed by 3 weeks of recovery (CPZ3+R3), and 3 weeks followed by 6 weeks of recovery (CPZ3+R6), along with their controls; and (**b**) 6 weeks (CPZ6), 6 weeks followed by 3 weeks of recovery (CPZ6+R3), and 6 weeks followed by 6 weeks of recovery (CPZ6+R6), along with their controls. Data are presented as mean staining intensity ± SEM; *n* = 6 per group. Statistical analysis was performed using a two-way ANOVA followed by Bonferroni’s multiple comparisons test. * *p* < 0.05, *** *p* < 0.001 versus control; ### *p* < 0.001 versus cuprizone. a.u.: arbitrary units.

The immunohistochemical quantification revealed a significant decrease in Apo D staining intensity in CPZ-treated mice at both 3 and 6 weeks. Notably, Apo D immunostaining is recovered in all groups subjected to recovery periods (Figure 10). 

**Figure 11:**
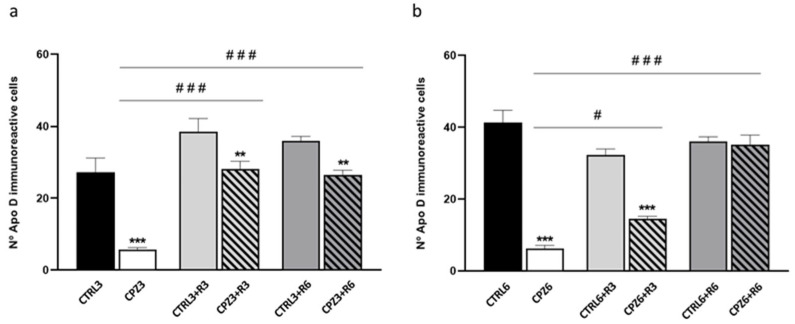
Quantification of the number of Apolipoprotein D (Apo D) immunopositive cells (oligodendrocytes) in the corpus callosum of mice treated with cuprizone for (**a**) 3 weeks (CPZ3), 3 weeks followed by 3 weeks of recovery (CPZ3+R3), 3 weeks followed by 6 weeks of recovery (CPZ3+R6), along with their respective controls; and (**b**) 6 weeks (CPZ6), 6 weeks followed by 3 weeks of recovery (CPZ6+R3), 6 weeks followed by 6 weeks of recovery (CPZ6+R6), along with their respective controls. Data are the mean ± SEM; *n* = 6 per group. Statistical analysis was performed using a two-way ANOVA followed by Bonferroni’s multiple comparisons test. ** *p* < 0.01, *** *p* < 0.001 versus control. # *p* < 0.05, ### *p* < 0.001 versus cuprizone.

Finally, to further assess the effect of CPZ treatment on Apo D expression, we also performed a cell count of Apo D-immunoreactive OLGs in the corpus callosum. As shown in Figure 11, CPZ3 and CPZ6 mice exhibited a significant reduction in Apo D-positive cells compared to controls. Consistent with previous observations, a marked restoration in the positive OLGs was noted after 3 and 6 weeks of recovery.

### 2.3. Clozapine Counteracts the Decrease in Apolipoprotein D Expression Caused by Cuprizone Treatment in the Corpus Callosum

**Figure 12:**
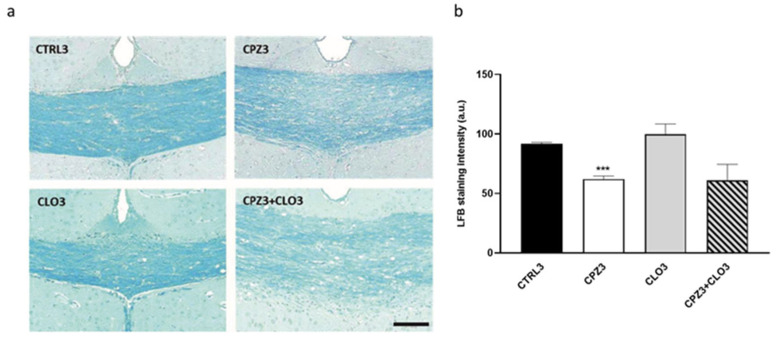
Representative images of coronal sections of the corpus callosum (0–0.3 mm Bregma level) stained with Luxol Fast Blue (LFB) from the brains of control mice and those treated for 3 weeks with cuprizone (CPZ), clozapine (CLO), or both. Scale bar: 100 µm (**a**). Densitometric quantification of LFB myelin staining. Data are presented as mean staining intensity ± SEM; *n* = 6 per group (**b**). Statistical analysis was performed using one-way ANOVA followed by Bonferroni’s multiple comparisons test. *** *p* < 0.001 versus control. a.u.: arbitrary units.

**Figure 13:**
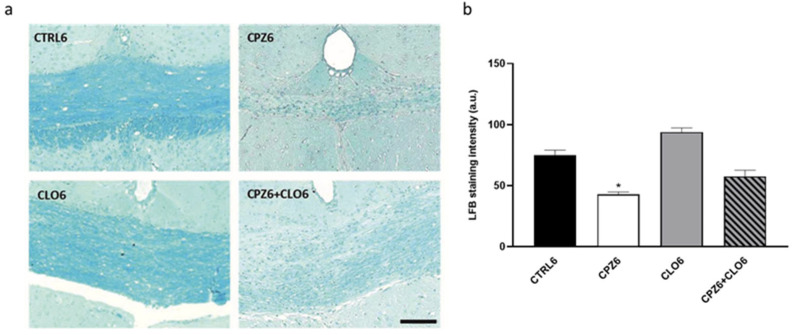
Representative images of coronal sections of the corpus callosum (0–0.3 mm Bregma level) stained with Luxol Fast Blue (LFB) from the brains of control mice and those treated for 6 weeks with cuprizone (CPZ), clozapine (CLO), or both. Scale bar: 100 µm (**a**). Densitometric quantification of LFB myelin staining. Data are presented as mean staining intensity ± SEM; *n* = 6 per group (**b**). Statistical analysis was performed using one-way ANOVA followed by Bonferroni’s multiple comparisons test. * *p* < 0.05 versus control. a.u.: arbitrary units.

Considering the previously reported CLO-induced increases in Apo D expression in certain white matter brain regions [65], we aimed to evaluate the effect of this antipsychotic on Apo D expression levels in our MS model. However, before addressing this, we initially evaluated the potential neuroprotective effect of CLO in CPZ-treated mice. To this end, animals were exposed to CLO, either alone or in combination with CPZ, for 3 or 6 weeks. As shown in the images and their corresponding densitometric analyses, CLO did not appear to counteract the myelin loss induced by CPZ in the corpus callosum of the studied mice (Figure 12 and Figure 13).

**Figure 14:**
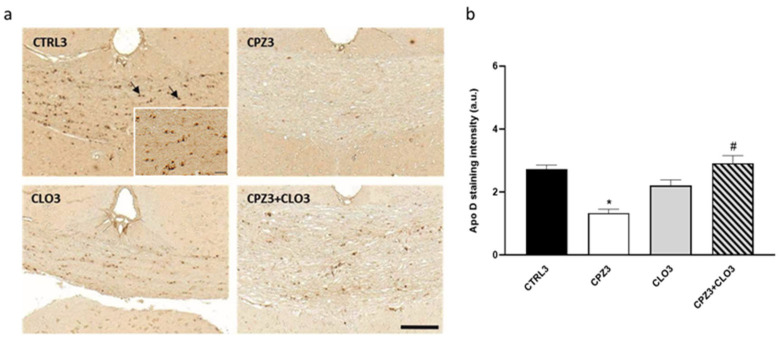
Representative images of Apolipoprotein D (Apo D) expression in coronal sections of the corpus callosum (0-0.3 mm Bregma level) from the brains of control mice and those treated for 3 weeks with (CPZ), clozapine (CLO), or both. Scale bar: 100 µm. Detail: 83×; scale bar 20 μm (**a**). Densitometric quantification of Apo D immunostaining. Data are presented as mean staining intensity ± SEM; *n* = 6 per group (**b**). Statistical analysis was performed using one-way ANOVA followed by Bonferroni’s multiple comparisons test. * *p* < 0.05 versus control; # *p* < 0.05 versus CPZ3. a.u.: arbitrary units.

**Figure 15:**
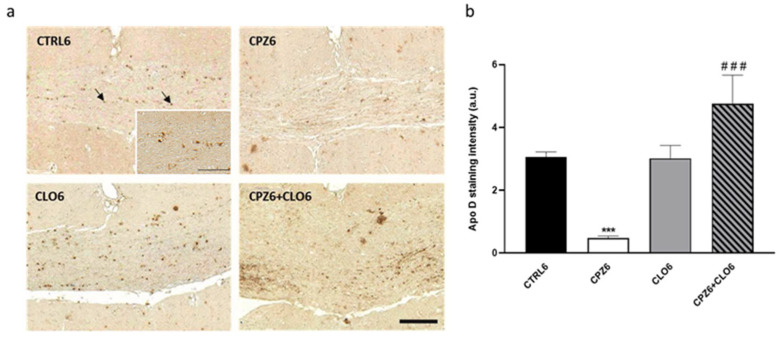
Representative images of Apolipoprotein D (Apo D) expression in coronal sections of the corpus callosum (0-0.3 mm Bregma level) from the brains of control mice and those treated for 6 weeks with (CPZ), clozapine (CLO), or both. Scale bar: 100 µm. Detail: 65×; scale bar 50 μm (**a**). Densitometric quantification of Apo D immunostaining. Data are presented as mean staining intensity ± SEM; *n* = 6 per group (**b**). Statistical analysis was performed using one-way ANOVA followed by Bonferroni’s multiple comparisons test. *** *p* < 0.001 versus control; ### *p* < 0.001 versus CPZ6. a.u.: arbitrary units.

Interestingly, immunohistochemical analysis for Apo D in brain sections from mice treated with CPZ, CLO, or both compounds simultaneously for 3 or 6 weeks demonstrated that CLO did not influence oligodendrocytic expression of this apolipoprotein. However, when CLO was administered together with CPZ, it attenuated the loss of Apo D expression caused by CPZ treatment; signal intensity in CPZ3- and CPZ6-treated mice was comparable to that of control animals (Figure 14 and Figure 15).

**Figure 16:**
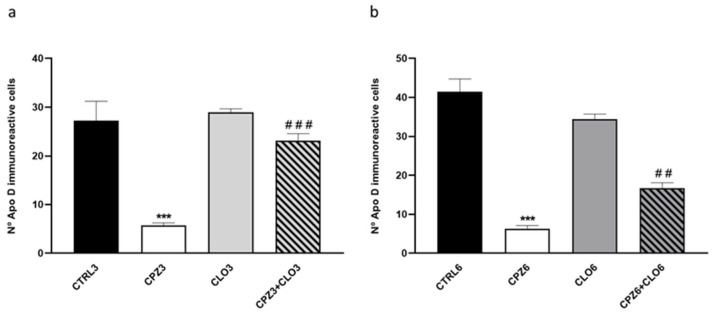
Quantification of the number of Apolipoprotein D (Apo D) immunopositive cells (oligodendrocytes) in the corpus callosum of mice: (**a**) control, treated with (CPZ), clozapine (CLO), or both for 3 weeks; and (**b**) control, treated with cuprizone CPZ, CLO, or both for 6 weeks. Data are the mean ± SEM; *n* = 6 per group. Statistical analysis was performed using one-way ANOVA followed by Bonferroni’s multiple comparisons test. *** *p* < 0.001 versus control. ## *p* < 0.01, ### *p* < 0.001 versus cuprizone.

Finally, counting the number of OLGs expressing Apo D revealed a significant decrease in Apo D-positive cells within the corpus callosum of mice treated with CPZ for 3 and 6 weeks compared to their respective controls. However, consistent with previously mentioned results, mice co-treated with CPZ and CLO maintained a constant number of positive OLGs in this brain region (Figure 16).

## 3. Discussion

Deciphering the molecular mechanisms that regulate the expression and function of Apo D under demyelinating conditions is a critical step toward advancing our understanding of CNS pathologies such as MS. Within this framework, experimental models of MS play a pivotal role, offering valuable tools to dissect the complex pathophysiological processes underlying demyelination and to evaluate potential therapeutic interventions at the preclinical level. Among available experimental systems [19,72], the CPZ-induced model of cytotoxic demyelination is particularly appreciated for reliably reproducing key CNS pathological features, including OLG loss, microglial activation, and spontaneous remyelination after toxin withdrawal [73,74]. Although it captures essential aspects of demyelination and partial remyelination, the model does not fully recapitulate the complex immune-mediated pathology of MS. Nevertheless, it remains a widely accepted and highly reproducible tool for studying OLG vulnerability, mitochondrial dysfunction, and the cellular mechanisms driving demyelination.

While demyelination remains the hallmark of CPZ toxicity, other measurable parameters such as hydrocephalus severity, brain weight, and motor cortex thickness are also indicative of CNS-level effects [75,76,77,78]. Although these variables have been individually investigated in previous studies, they had not been comprehensively assessed in combination prior to the present work. Thus, MRI analysis revealed hyperintense regions in the brain as early as week 3, suggestive of parenchymal edema potentially caused by CSF accumulation and increased extracellular space due to OLG damage [79,80]. By week 6, these alterations progressed to aqueductal stenosis and hydrocephalus, a phenomenon rarely reported in the literature [81,82], which were confirmed using Evans’ ratio [83,84]. Although hydrocephalus is typically associated with higher CPZ doses [85,86], our findings demonstrate that it can also occur at the standard 0.25% (*w*/*w*). As expected, brain weight increased in CPZ-treated mice compared to controls at both 3 and 6 weeks of treatment, and this effect was reversible. However, it is important to note that increased brain weight does not necessarily reflect the presence or severity of hydrocephalus, as CSF has a lower specific gravity than neural tissue. Moreover, tissue damage and parenchymal loss associated with elevated intracranial pressure may offset any apparent mass gain due to fluid accumulation [87].

Traditionally, MS has been considered a disease primarily affecting white matter. However, increasing evidence highlights the involvement of cortical and hippocampal gray matter demyelination, particularly in progressive forms of the disease, where it significantly contributes to irreversible neurological disability [88,89]. The CPZ model is one of the few experimental paradigms that reliably induces gray matter damage, particularly in the frontal cortex, hippocampus, and deep brain nuclei, with notable changes emerging by week 4 of treatment [90,91,92,93]. In this study, thinning of the motor cortex was already apparent after 3 weeks of CPZ exposure and became more pronounced following 6 weeks. Since the CPZ-induced cytotoxicity model does not typically result in neuronal loss [74], the reduction in cortical thickness is most likely attributable to demyelination within the cortical gray matter [73].

The histological and imaging findings of this work confirmed a time-dependent myelin loss in the corpus callosum of the CPZ-treated mice, consistent with previous studies [71,94,95]. Remarkably, effective remyelination within this region, accompanied by recovery of brain volume and motor cortex thickness, was observed once the treatment with CPZ ceased. These observations agree with previous studies demonstrating remyelination through electron microscopy and immunohistochemistry following CPZ withdrawal [96]. Notably, gray matter remyelination has also been reported post-treatment [97]. Nevertheless, edema and hydrocephalus may persist beyond the treatment period, as parenchymal alterations induced by CPZ appear to be only partially reversible [98,99].

Given its usefulness for studying the pathophysiology of MS and for evaluating potential remyelinating or neuroprotective therapies, the CPZ-induced model enabled us to investigate the role of Apo D within this physiologically relevant context by assessing its expression in the corpus callosum following CPZ administration. Our results reveal a time-dependent decrease in Apo D expression at 3 and 6 weeks of treatment, which closely paralleled the extent of demyelination observed. This reduction was further reflected in the diminished presence of Apo D-immunoreactive OLGs. Interestingly, these effects were reversed following the cessation of CPZ treatment which suggests a link between Apo D expression, myelin content, and the integrity of OLG populations. To confirm the oligodendrocytic nature of the Apo D-expressing cells, we conducted additional double immunohistochemistry using Olig-2 as a marker for OLGs, and GFAP for astrocytes. Colocalization with Olig-2 and the absence of signal overlap with GFAP strongly support the specificity of Apo D expression in OLGs under our experimental conditions.

The loss of myelin sheaths surrounding axons is closely associated with OLG degeneration, a well-established effect of CPZ exposure and a hallmark of MS pathology [98,100,101,102]. Since both mature OLGs and OPCs are key sources of Apo D during CNS development and in the adult brain [16,53], our findings support the hypothesis that CPZ-induced OLG damage underlies the observed reduction in Apo D levels and myelin content. Notably, following CPZ withdrawal, we observed a restoration in the amount of myelin, Apo D expression, and the number of Apo D-positive OLGs. This recovery suggests that surviving OPCs may differentiate into mature OLGs capable of synthesizing Apo D and generating new myelin sheaths, consistent with previous reports [48,103]. Similar Apo D expression patterns have been reported in post-mortem brain samples from MS patients; the apolipoprotein levels were reduced in demyelinated lesions, particularly in inactive plaques, and was restored in remyelinating areas [16]. These observations suggest a potential role for Apo D in spontaneous remyelination, contributing to homeostatic and regenerative processes rather than directly preventing demyelination. This interpretation is consistent with its previously proposed functions in myelin dynamics [17,54,104]. Nevertheless, although our data currently reflect a reduction in the number of Apo D-positive cells, which may result from transcriptional downregulation or selective loss of these cells, the interpretation of the labeling results could be strengthened in future studies through the incorporation of techniques such as ultrastructural confirmation by electron microscopy and gene expression analyses, which will be essential to clarify this distinction.

Numerous studies conducted by independent research groups worldwide have demonstrated the relevance of Apo D in a variety of neurodegenerative disorders where it is recognized for its neuroprotective, anti-inflammatory, and antioxidant properties [41,60,105,106]. Specifically, it has been shown to mitigate oxidative stress, prevent glial and neuronal apoptosis, and contribute to the repair of damaged neural tissue [67,107,108,109]. Some preclinical and clinical studies have documented the overexpression of Apo D in glial cells during demyelination/remyelination processes, such as those occurring in this pathology [16,60]. In the PNS, its expression is upregulated in Schwann cells and macrophages following sciatic nerve injury, promoting motor function recovery, and regulating the timing and magnitude of the inflammatory response [49,67,68,69]. In fact, it is shown that Apo D deficiency compromises myelin compaction and clearance following injury, leading to delayed axonal regeneration and remyelination [49,67]. In the CNS, Apo D expression increases with age and in several pathological conditions [51,110,111]. Interestingly, elevated Apo D mRNA levels have been observed in mature OLGs and reactive astrocytes in ischemic rat brains, particularly in white matter and peri-lesional areas, suggesting a potential neuroprotective role in promoting remyelination during recovery [48]. In the context of MS, demyelination, axonal regeneration, and remyelination are dynamic processes that require substantial lipid synthesis to support the formation of new myelin sheaths. Apo D, a key glial protein involved in myelin remodeling, shows decreased expression in the CPZ-induced murine model of MS, likely due to both a reduction in OLG population and impaired expression in surviving cells affected by CPZ toxicity [112]. Also, inflammatory cytokines like IL-1 and IL-12, released by activated microglia and astrocytes, may further disrupt Apo D expression [29,113]. In response to injury, OPCs work to restore lost OLGs through proliferation and differentiation driven by glial-derived signals. In this context, Apo D may facilitate regeneration by modulating cell migration and acting as a neurotrophic factor, promoting neurite outgrowth and synaptogenesis [114,115,116,117].

Finally, considering the proposed involvement of Apo D in axonal functional integrity, we aimed to modulate its expression using the atypical antipsychotic CLO. Several studies have demonstrated that CLO can modulate microglial activation, reduce oxidative stress, and influence astrocytic responses, thereby attenuating processes directly implicated in demyelination and OLG loss [118,119,120]. These mechanisms are particularly relevant in the CPZ model, where mitochondrial dysfunction, oxidative stress, and glial reactivity are major contributors to cytotoxicity [73,74,112]. Thus, the use of CLO in this paradigm provides not only a pharmacological approach to mitigate CPZ-induced damage but also enhances the translational value of the model by addressing cellular and molecular mechanisms that overlap with those occurring in MS.

In our study, the co-administration of CLO with CPZ in mice did not reverse myelin loss in the corpus callosum. While previous studies, albeit limited, have reported improvements in motor function and reductions in glial reactivity following CLO administration after CPZ treatment [119,121,122], these effects occurred in the absence of significant remyelination [119,121]. Consistent with our previous in vitro findings [123], we observed no changes in Apo D expression under physiological conditions after CLO treatment. Remarkably, while CLO effectively counteracted the CPZ-induced suppression of oligodendrocytic Apo D expression, this effect did not translate into any improvement in myelin content.

The neuroprotective effect of CLO has been well established. Previous studies have shown that CLO benefits OLG in culture by enhancing mitochondrial energy supply by improving oxidative phosphorylation efficiency and stimulating the synthesis of myelin lipids such as galactocerebrosides [124]. Similarly, Xu et al. (2013) reported that the inhibition of OLG proliferation and maturation induced by CPZ in cell culture studies was partially reversed by treatment with CLO or quetiapine, another atypical antipsychotic [125]. In this context, and based on our previously published in vitro data, Apo D may play a contributory role in the protective actions of CLO [123]. This hypothesis is further supported by animal studies suggesting that effects of CLO under pathological conditions are associated with the protective functions of Apo D, including: (i) binding hydrophobic ligands, (ii) minimizing their release, (iii) preventing lipid peroxidation, and (iv) stabilizing plasma membranes [65,126]. Despite the presumed role of Apo D as a mediator of certain CLO-related neuroprotective effects, the precise molecular mechanisms remain unclear [29]. Apo D is known to be involved in various cellular stress responses; however, its role appears to be highly dependent on both the pathological context and its cell-type-specific expression patterns [50,64,127,128,129]. CLO has been shown to influence intracellular signaling pathways such as PI3K/Akt and MAPK/ERK, which regulate cell survival, oxidative stress responses, and the expression of lipid-binding proteins [130,131]. In this regard, Apo D is typically upregulated in response to cellular stress, oxidative injury, or inflammation, acting as a neuroprotective molecule through its involvement in lipid transport, membrane stabilization, and antioxidant defense [45,65,126]. Therefore, CLO may increase Apo D expression both directly, via transcriptional activation of stress-response genes, and indirectly, by promoting a cellular environment conducive to neuroprotective gene expression. In our model, a longer duration of CLO treatment may be required for Apo D to exert a functional impact on CPZ-induced demyelination and subsequent remyelination. Future studies employing pre- and post-treatment paradigms will be critical to further elucidate these interactions and to identify the specific molecular mediators and transcription factors involved in this regulatory mechanism. Moreover, although CLO is not a standard therapy for MS, its pharmacological profile highlights relevant pathways that could potentially be targeted by safer and more specific compounds in the future. In this context, Apo D expression may serve as a biomarker to monitor glial activity and oxidative stress responses, providing valuable insights into treatment efficacy.

While CLO provided a useful pharmacological tool in our study, it is important to consider whether current MS therapies may also affect Apo D expression or function. Indeed, several DMTs target molecular cascades associated with oxidative stress and lipid handling [132,133,134]. For example, dimethyl fumarate activates the Nrf2 pathway, promoting antioxidant defenses [135], whereas fingolimod modulates sphingolipid signaling through S1P receptors [136,137], a process that could intersect with Apo D-mediated lipid transport. Direct evidence for Apo D modulation by these drugs is still scarce, our results suggest that Apo D may represent a common downstream effector of different therapeutic strategies.

In conclusion, this work reinforces the neuroprotective role of Apo D in the context of myelin dynamics and demyelinating pathology, providing a solid foundation for future studies aimed at identifying novel therapeutic targets, particularly for progressive forms of MS and related disorders. A thorough and systematic investigation of Apo D expression and function in experimental models of MS will be essential to clarify its mechanistic involvement in demyelination and remyelination and to fully evaluate its translational potential.

## 4. Materials and Methods

### 4.1. Animal Model and Experimental Design

In this study, a total of 96 male C57BL/6 mice (8 weeks old, 20–25 g) were obtained from the Animal Facility of the Scientific and Technical Services (SCTs) at the University of Oviedo. Mice were housed in positive pressure-ventilated racks at 25 ± 1 °C with a 12 h light/dark cycle, fed ad libitum with a standard grounded chow, and allowed free access to filtered and UV-irradiated water. All animal care and experimental procedures were in accordance with European and Spanish regulations (86/609/CEE; RD1201/2005) and were approved by the Research Ethics Committee of the University of Oviedo and the "Consejería de Agroganadería y Recursos Autóctonos del Principado" (PROAE14/2016; 04/04/2016; approved on 4 April 2016).

Before the experiments, animals were acclimated to the experimental environment for one week. During this period, mice were individually housed in acrylic cages and fed ground chow (Teklad 2014s, EnVigo) under the same standard conditions previously described. To establish the CPZ-induced demyelination model, animals were randomly assigned to different groups: (1) Control groups (CTRL, *n* = 36), fed a standard diet; (2) Cuprizone-treated group (CPZ3, *n* = 6), receiving a 0.25% (*w*/*w*) CPZ (bis(cyclohexanone)oxaldihydrazone, C9012-25G, Sigma-Aldrich, St. Louis, MO, USA) enriched diet for 3 weeks to induce demyelination [21,23]; (3) CPZ6 (*n* = 6), treated with CPZ for 6 weeks; (4) Recovery group (CPZ3+R3, *n* = 6), in which CPZ administration was discontinued after 3 weeks, allowing spontaneous remyelination for an additional 3 weeks; (4) CPZ3+R6 (*n* = 6), treated with CPZ for 3 weeks followed by a 6-week recovery period; (5) CPZ6+R3 (*n* = 6), treated with CPZ for 6 weeks followed by a 3-week recovery period; and (6) CPZ6+R6 (*n* = 6), treated with CPZ for 6 weeks followed by a 6-week recovery period.

Additionally, mice in the CLO-treated groups received 10 mg/kg/day CLO (C6305, Sigma-Aldrich, St. Louis, MO, USA) dissolved in drinking water, prepared fresh daily, based on the protocol previously described [106]. The CLO groups included CLO3, CLO6, CPZ3+CLO3, and CPZ6+CLO6.

The mice were weighed every two days, and the water consumption was measured. Animals were sacrificed next day after completion of treatments.

### 4.2. Magnetic Resonance Imaging

To evaluate changes in myelin integrity in vivo, MRI scans were performed on a 3 Tesla small-animal MRI system (RM MRS 3000, MR Solutions, Surrey, UK) at the Preclinical Imaging Unit of the SCTs. Mice were anesthetized with 2–2.5% isoflurane inhalation (mg/kg) and placed in a stereotaxic holder. T2-weighted images were acquired at baseline and endpoint. Regions of interest were selected, and brain volume, ventricular volume, motor cortex thickness, and corpus callosum density were measured using ImageJ version 1.54p. The Evans index, defined as the ratio of the maximum width of the lateral ventricles to the maximum width of the brain, was used to evaluate hydrocephalus, with values of ≥0.3 indicating pathological dilation.

### 4.3. Tissue Processing and Histochemical Staining

Tissue samples of the brains were obtained after intracardiac perfusion with 4% paraformaldehyde (PFA) in phosphate buffer, removed from the skull, post-fixed for 12 to 18 h in 4% PFA and then embedded in paraffin. Transversal sections of 5 μm thick at (0–0.3) Bregma level were obtained, mounted on “SuperFrost Plus” (Menzel-Glasse), and incubated in a household microwave oven. Microwave treatment involves completely blocking contaminating staining in the double-labeling technique using primary antibodies from the same species and the same secondary antibody. Then, incubation with a specific polyclonal antibody against Olig-2 (AB9610, Millipore, Burlington, MA, USA) was carried out overnight at 4 °C. After several washes in PBS, sections were incubated for 30 min at room temperature in biotinylated horse universal antibody diluted 1:40, and subsequently with streptavidin Alexa Fluor^®^ 550 conjugate (1:500; S2138, Invitrogen, Paisley, Scotland, UK). Finally, cells were washed in distilled water, dehydrated, cleared in eucalyptol and mounted with Fluoromount^™^ (F4680, Sigma-Aldrich, St. Louis, MO, USA).

The sections were observed using an Epi-Fl Nikon Eclipse E400 microscope (Nikon, Minato-ku, Tokyo, Japan) equipped with Plan Fluor objectives, and images were recorded using a digital camera (20×; NikonDN-100, Nikon, Minato-ku, Tokyo, Japan). Final images were obtained through the digital superposition of the corresponding DAB Apo D signal and red fluorescence Olig-2 signal images of the same sections. The positive signal of each image was selected according to the method of Navarro et al. (2008) [138]; DAB signals were converted to green and saved as an RGB image. Merged images show the Olig-2 fluorescence signal in red and the DAB label in green; the yellow color indicates the superposition of red and green colors.

### 4.4. Immunohistochemistry for Apolipoprotein D and GFAP

For the same purpose described above, adjacent serial sections of the corpus callosum were processed for double immunohistochemistry with Apo D and GFAP, following the protocol detailed below. To this end, sections were permeabilized with 0.1% Triton X for 5 min, washed in distilled water, treated with 3% H_2_O_2_ for 5 min to eliminate endogenous peroxidase activity, washed in distilled water again, and treated with PBS for 2 min. Non-specific binding was blocked by incubation with 1% BSA for 30 min at room temperature. Incubation with a specific rabbit antibody against human Apo D (1:2000), was carried out overnight in a humid chamber at 4 °C. After several washes in PBS, sections were incubated 30 min at room temperature using a biotinylated horse universal antibody diluted 1:40, followed by a 30 min incubation with peroxidase-labeled extravidin. The peroxidase activity was visualized with 0.05% DAB in 50 mM Tris buffer pH 7.6, containing 0.04% H_2_O_2_ (33%). Following the detection of Apo D expression using DAB, slides were rinsed in PBS, placed in a plastic Coplin jar filled with 0.01 M sodium citrate buffer (pH 6), and incubated in a household microwave oven. Then, incubation with a specific monoclonal antibody against GFAP (1:200; G-3893, Sigma BioSciences, St. Louis, MO, USA) was carried out overnight at 4 °C. After several washes in PBS, sections were incubated for 30 min at room temperature in biotinylated horse universal antibody diluted 1:40, and were subsequently incubated with streptavidin Alexa Fluor^®^ 550 conjugate (1:500). Finally, cells were washed in distilled water, dehydrated, cleared in eucalyptol and mounted with Fluoromount^™^.

Image acquisition and processing for Apo D and GFAP-stained sections followed the same protocol previously described.

### 4.5. Immunohistochemical Staining Quantification

Sections were observed using an Epi-Fl Nikon Eclipse E400 microscope equipped with Plan-Fluor objectives, and images were recorded by a digital camera. For the quantification of Apo D presence and LFB stain in the central area of the corpus callosum, the chromogenic signal was selected with Adobe Photoshop CS 8.0.1 (Adobe Systems Inc., San Jose, CA, USA) and quantified with ImageJ 1.37c (National Institutes of Health, Bethesda, MD, USA) software according to a procedure developed by our group [139]. Six random regions per case were photographed using a 20× lens.

The number of Apo D positive cells was also counted. In this sense, a cell was considered positive if it exhibited distinct Apo D immunoreactivity above background levels. Quantification was performed in three randomly selected fields per sample (or animal) at 25× magnification. When there was any ambiguity regarding the positivity of a cell, the magnification was increased to 60× to ensure accurate identification. Counts were repeated three times to ensure consistency and reproducibility.

### 4.6. Data Analysis

The data in the graphs are presented as the mean ± SEM. Statistical analysis was performed using GraphPad Prism version 8 (San Diego, CA, USA). A one or two-way ANOVA followed by Bonferroni’s post hoc test were used for multiple comparisons. Differences were considered significant when *p* < 0.05. 

## Data Availability

The datasets generated and/or analyzed during the current study are not publicly available due to privacy/ethical restrictions, but are available from the corresponding author upon reasonable request.

## Supplementary Materials

The following supporting information can be downloaded at: https://www.mdpi.com/article/10.3390/ijms26178692/s1.

## Abbreviations

The following abbreviations are used in this manuscript:

AD	Alzheimer’s disease
Apo D	apolipoprotein D
BSA	bovine serum albumin
CLO	clozapine
CNS	central nervous system
CPZ	cuprizone
CSF	cerebrospinal fluid
DAB	diaminobenzidine
LFB	luxol fast blue
MRI	magnetic resonance imaging
MS	multiple sclerosis
NS	nervous system
NSCs	neural stem cells
OLG	oligodendrocyte
OPCs	oligodendroglial progenitor cells
PBS	phosphate-buffer saline
PFA	paraformaldehyde
PNS	peripheral nervous system
RRMS	relapsing-remitting MS
SCTs	scientific and technical services
