# Supplementary figures and images for: Apolipoprotein D Expression Dynamics During Cuprizone-Induced Demyelination and Remyelination in a Mouse Model of Multiple Sclerosis
Source: Int J Mol Sci. 2025 Sep 6;26(17):8692. doi: 10.3390/ijms26178692 (PMC12429407; doi:10.3390/ijms26178692)

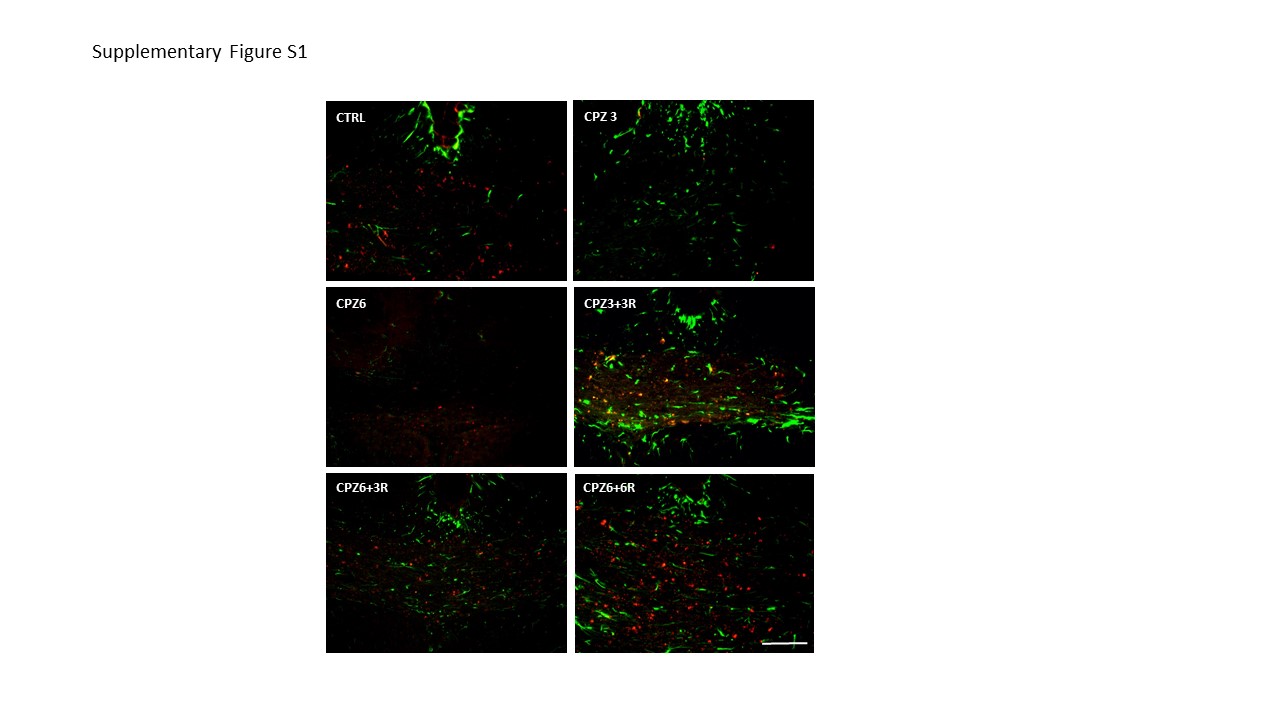

Supplement: Supplementary file 1 [file ijms-26-08692-s001.zip › ijms-3829016-supplementary.JPG]
